# Should oxidosqualene cyclase in the cholesterol biosynthetic pathway be considered an anti-cancer target?

**DOI:** 10.3389/fcell.2022.1081151

**Published:** 2022-12-13

**Authors:** Slight SH, Hyder SM

**Affiliations:** Department of Biomedical Sciences and Dalton Cardiovascular Research Center, University of Missouri, Columbia, MO, United States

**Keywords:** cholesterol biosynthesis, cancer, estrogen receptor alpha, estrogen receptor beta, oxidosqualene cyclase

## Introduction

Many types of cancer are associated with aberrant cholesterol biosynthesis. There is increasing evidence that dysregulation of cholesterol synthesis within tumor cells promotes a milieu that is rich in cholesterol and is conducive to cellular proliferation, tumor progression and ultimately metastasis. The cholesterol metabolite 27-hydroxycholesterol has been specifically implicated in the progression of breast cancer (Nelson et al., 2013). Studies show that inhibiting the local production of cholesterol can hinder cancer development.

Cholesterol is an essential component of cellular membranes as well as being the precursor of a variety of steroid hormones. Its synthesis occurs through a sequence of complex reactions, beginning with the precursor acetyl-CoA, which is converted to hydroxyl methylglutaryl-CoA (HMG-CoA). The next step in the process, conversion of HMG-CoA to mevalonate, is catalyzed by the enzyme HMG-CoA reductase, which is targeted clinically by a variety of statin inhibitors to lower levels of circulating cholesterol and prevent the associated cardiovascular pathologies. Statins are anti-proliferative and consequently their effects against a variety of cancers have been studied, often with promising results. Commonly used drugs such as atorvastatin and simvastatin have been found to suppress cancers of the breast, lung, and ovary, among others (Barbalata et al., 2020). Statins, however, especially when given at high enough levels to be antitumorigenic, are linked to a number of undesirable side-effects due to their lowering of isoprenoid levels, causing defective posttranslational modification of membrane proteins and impaired membrane structure and function (McTaggart, 2006). Statins are also associated with an increase in type 2 diabetes (Ward et al., 2019). Because of the risks associated with statins, researchers have sought alternative means by which to disrupt cholesterol synthesis, that avoid the detrimental effects of statins.

## New targets to lower cholesterol biosynthesis

A few steps downstream from HMG-CoA reductase in the biosynthesis of cholesterol, the enzyme 2,3-oxidosqualene cyclase (OSC) converts 2,3-monoepoxysqualene to lanosterol, from which cholesterol is ultimately derived. OSC inhibition is a potentially fruitful area of research with respect to lowering cholesterol levels, and studies using OSC inhibitors indeed show promise as a means of slowing or reversing a variety of cancers, without the harmful side-effects of statins. One such inhibitor is RO 48-8071 (4’-[6-(allylmethylamino) hexyloxy]-4-bromo-2′-fluorobenzophenone fumarate) (RO). RO has been shown to effectively suppress several different types of cancer, including cancers of the breast, pancreas, prostate, and ovary. Although a variety of different OSC inhibitors have been studied, RO appears to be particularly promising, hence our discussion will concentrate on this particular inhibitor.

RO was developed by Hoffman-La Roche and its effectiveness at lowering plasma cholesterol levels studied in a variety of animal models (Morand et al., 1997). Our interest in the inhibitor occurred serendipitously following a study, using an inverse-docking approach, to identify novel small molecule anti-cancer drugs (Grinter et al., 2011). This led to an investigation at both the cellular level, and *in vivo*, using mouse xenograft models, into the effects of RO on breast cancer. RO induced apoptosis of tumor vessels in these mouse models, and quashed metastasis. Subsequent studies, using a spontaneous mouse model of pancreatic cancer (RIP-Tag2), and metastatic murine models of human colon carcinoma (HCT116) and pancreatic adenocarcinoma (HPAF-II), showed that RO suppressed angiogenesis, thereby reducing tumor vascularization (Maione et al., 2015).

In studies aimed at developing novel therapeutic techniques to combat hormone-dependent breast cancer, we found that RO decreased the expression of progesterone receptors in BT-474 and T-47D human breast cancer cells, and, furthermore, reduced levels of progestin-induced markers of cancer stem cells (CSCs), such as aldehyde dehydrogenase (ALDH), which are a hallmark of aggressive tumor growth, metastasis, and reappearance of cancer (Liang et al., 2017). RO also reduced expression of estrogen receptor α (ERα), which is known to promote proliferation of hormone-dependent breast cancer cells, while inducing ERβ expression (Liang et al., 2014). This latter observation is particularly exciting because ERβ is known to exert anti-proliferative effects in breast cancer cells. Studies conducted using a nude mouse model of BT-474 cell-derived xenografts grown in an estrogen-rich environment demonstrated that RO suppressed tumor growth *in vivo.* Immunohistochemical analysis of tumors collected at the end of the study showed that levels of ERα were also reduced *in vivo,* though in contrast to our observations using cultured BT-474 cells*,* up-regulation of ERβ was not as obvious. It is likely that this was due to tumors being collected at the experiment’s endpoint, by which time tumor cells expressing high levels of ERβ had already undergone apoptosis. By lowering the cellular ERα/ERβ ratio, RO reduces tumor cell proliferation.

In recent years there has been a search for naturally occurring plant compounds with anti-cancer properties. Our laboratory has studied several such agents and used them in combination with chemotherapeutic drugs that are often highly toxic, in order to develop novel, less toxic treatment methods. Recently, we investigated the combined effects of RO with one such compound, liquiritigenin (LQ), a flavonoid found in licorice that is also an ERβ agonist (Liang et al., 2022). A combination of RO and LQ significantly reduced the viability of cultured MCF-7 and BT-474 breast cancer cells compared with cells treated with either compound alone. Furthermore, growth of BT-474 derived tumor xenografts was inhibited *in vivo* and although both compounds reduced expression of ERα and markers of angiogenesis, while inducing ERβ expression and increasing apoptosis, a combination of the two enhanced these effects significantly over either agent alone.

A similar picture emerges when the effects of RO on prostate cancer are examined. In the early stages of prostate cancer, prostate cells express androgen receptors (AR) and their growth is hormone-dependent and treatable using anti-androgens. However, despite treatment, drug-resistant tumors often emerge that are classified as castration-resistant, despite AR still being involved in their growth (Shen et al., 2012). We conducted studies to investigate the effects of RO on both types of prostate cancer cells. When hormone-dependent (LNCaP) and castration-resistant (PC-3 and DU145) prostate cancer cells were exposed to RO, their viability was markedly reduced (Liang et al., 2016a). Normal prostate cells, grown in the presence of RO were, however, unaffected, suggesting little or no toxicity to normal cells. RO induced apoptosis in both hormone-dependent and castration-resistant prostate cancer cells and reduced levels of androgen receptors (AR), which are pro-proliferative and vital to the development of both types of prostate cancer cells. RO increased the levels of ERβ in both hormone-dependent LNCaP and castration-resistant PC-3 prostate cancer cells, demonstrating an anti-proliferative effect similar to that observed in breast cancer cells. Incubation of PC-3 cells with diarylpropionitrile (DPN), an ERβ agonist that stimulates ERβ activity, potentiated the effects of RO by reducing cell viability synergistically. The inhibitory effects of RO on prostate cancer *in vivo* were comparable with those observed in cultured cells. Castration-resistant PC-3 cells were injected into nude mice to create xenograft tumors which were allowed to develop for 6 weeks prior to administration of RO. Compared with control animals that did not receive the inhibitor, tumor growth was markedly suppressed, and, in some animals, tumors were completely eradicated by RO.

Ovarian cancer cells are known to express enzymes that are involved in cholesterol biosynthesis, including OSC (Zhao et al., 2021). Since we and others have shown RO to be effective in reducing the viability of a variety of cancer cells, and suppressing tumor growth, we conducted studies aimed at determining its effectiveness against epithelial ovarian cancer (EOC) (Liang et al., 2016b). OVCAR-3 (high grade serous) and SK-OV-3 (non-high grade serous) cells, exposed to RO in pharmacological and nanomolar levels, showed a significant loss of viability, whereas RO had no effect on normal ovarian cells. Furthermore, RO reduced the acquisition of stem-cell like properties by SK-OV-3 cells, as demonstrated by inhibition of ALDH activity. As in other types of cancer, RO effectively suppressed the growth of subcutaneous tumors derived from SK-OV-3 EOC cells in our well-developed nude mouse xenograft model. However, following an initial response to RO, tumors became resistant and started to grow again, suggesting that combination therapy using a combination of RO and additional chemotherapeutic agents might be necessary to fully control ovarian cancer growth *in vivo.*

The effects of RO on the viability of the pancreatic ductal adenocarcinoma cell line PANC-1 are in accordance with its effects in other types of cancer. RO was shown to reduce PANC-1 viability *in vitro* in a time-dependent manner, by inhibiting the cell cycle at the G_1_S phase (Ding et al., 2021). In studies to ascertain which cell cycle related genes might be affected by RO, it was found that expression of both cyclin B1 and cyclin E were reduced by RO, while p27 expression was increased. RO also affected the phosphorylation levels of ERK and JNK in PANC-1 cells, which suggests it may thereby exert certain anti-tumor effects by regulating apoptosis, angiogenesis, and metastasis. In xenograft studies in nude mice bearing PANC-1 derived tumors, it was shown that RO reduced tumor growth and size compared with controls. Immunohistochemical staining of tumor sections demonstrated lower levels of Ki67 staining in response to RO, confirming inhibition of PANC-1 cell proliferation *in vivo.*

## Conclusion

Although statins are currently the drugs of choice for inhibiting the production of, and lowering circulating levels of cholesterol, to prevent the cardiovascular pathologies associated with high cholesterol, there is evidence in animal models that OSC inhibitors such as RO offer a viable, non-toxic alternative that avoids the negative consequences of statins (Morand et al., 1997). The anti-cancer effects of RO have yet to be fully realized; however, by inhibiting angiogenesis, reducing tumor vascularization, and suppressing metastasis, RO appears to exert its effects consistently against tumors of different origin, suggesting it might be beneficial against a wide variety of cancers. It remains to be investigated whether RO can exert anti-inflammatory effects and/or attract immune cells to destroy tumors. The anti-tumor effects of RO against various tumor types suggest the latter as possible mechanisms for anti-cancer effects of RO. Based on our observations of its potency against breast cancer, we believe that RO could be especially advantageous to postmenopausal women who are at risk for developing the disease, particularly those undergoing combination hormone replacement therapy (HRT) containing both estrogen and progestin, which is associated with an increased risk of breast disease. We propose that clinical studies to assess the effectiveness of RO in a variety of medical settings, including breast, prostate, and ovarian cancer, be initiated, with a view to providing physicians with a new, non-toxic agent with which to combat these diseases.
